# Empowering Environmental Justice Decision Makers: Increasing Educational Resources for U.S. Environmental Protection Agency’s Mapping Tools

**DOI:** 10.1089/env.2021.0037

**Published:** 2021-10-04

**Authors:** Jenna M. Hartley, Stacey Lobatos, Jessica L. Daniel, Tai Lung

**Affiliations:** ORISE Participant on the EnviroAtlas Team, U.S. Environmental Protection Agency, Office of Research & Development, Durham, North Carolina, USA, as well as a PhD student in the Environmental Education Lab at North Carolina State University, Raleigh, North Carolina, USA; Training and Curriculum Coordinator at the Office of Environmental Justice, U.S. Environmental Protection Agency, Washington, District of Columbia, USA.; EnviroAtlas Outreach, Communications, and Stakeholder Engagement Lead at the Office of Research & Development, U.S. Environmental Protection Agency, Durham, North Carolina, USA.; EJSCREEN Project Lead at the Office of Environmental Justice, U.S. Environmental Protection Agency, Washington, District of Columbia, USA.

**Keywords:** activism, children and the environment, citizen advocates, EPA, science and technology, social justice

## Abstract

Policy development and subsequent action occur at all levels of government with various opportunities for input from nongovernmental organizations and individual citizens. U.S. Environmental Protection Agency (U.S. EPA) mapping tools such as EJSCREEN and EnviroAtlas incorporate environmental and demographic data to inform local decision makers on issues and policies in their communities by putting data, resources, and information in the hands of citizens. However, these tools are only as powerful as their reach, especially when it comes to communities with environmental justice (EJ) concerns. In this article, we present the development of an EJ educational case study based on a collaboration between U.S. EPA’s Office of Environmental Justice and Office of Research and Development (ORD) and the Chesapeake Bay Foundation. Through the EPA Case Study, we state the need for EJ-focused educational materials that leverage the U.S. EPA’s mapping tools, describe the development and application of the EPA Case Study, and share lessons learned to help inform future EJ educational materials development. We conclude with a literature-backed call to action for continued federal support in the development of tools and educational materials to inform decision making that has the potential galvanize policy change.

## INTRODUCTION

U.S. Environmental Protection Agency (U.S. EPA) mapping tools such as EJSCREEN and EnviroAtlas incorporate environmental and demographic data to inform local decision makers on important issues and policies in their communities. These tools put data, resources, and information in the hands of citizens young and old to empower communities and influence policy. While powerful, they have an opportunity to do more to bridge the gap between science and the public, especially when it comes to communities with environmental justice (EJ) concerns.^1^ By providing resources that demonstrate how data and information can be used at all levels, from youth education to formal policy, EPA and other federal agencies can better equip citizens with the skills they need to use federal tools and help inform the decisions they make on behalf of their communities.^2^

**FIG. 1.**
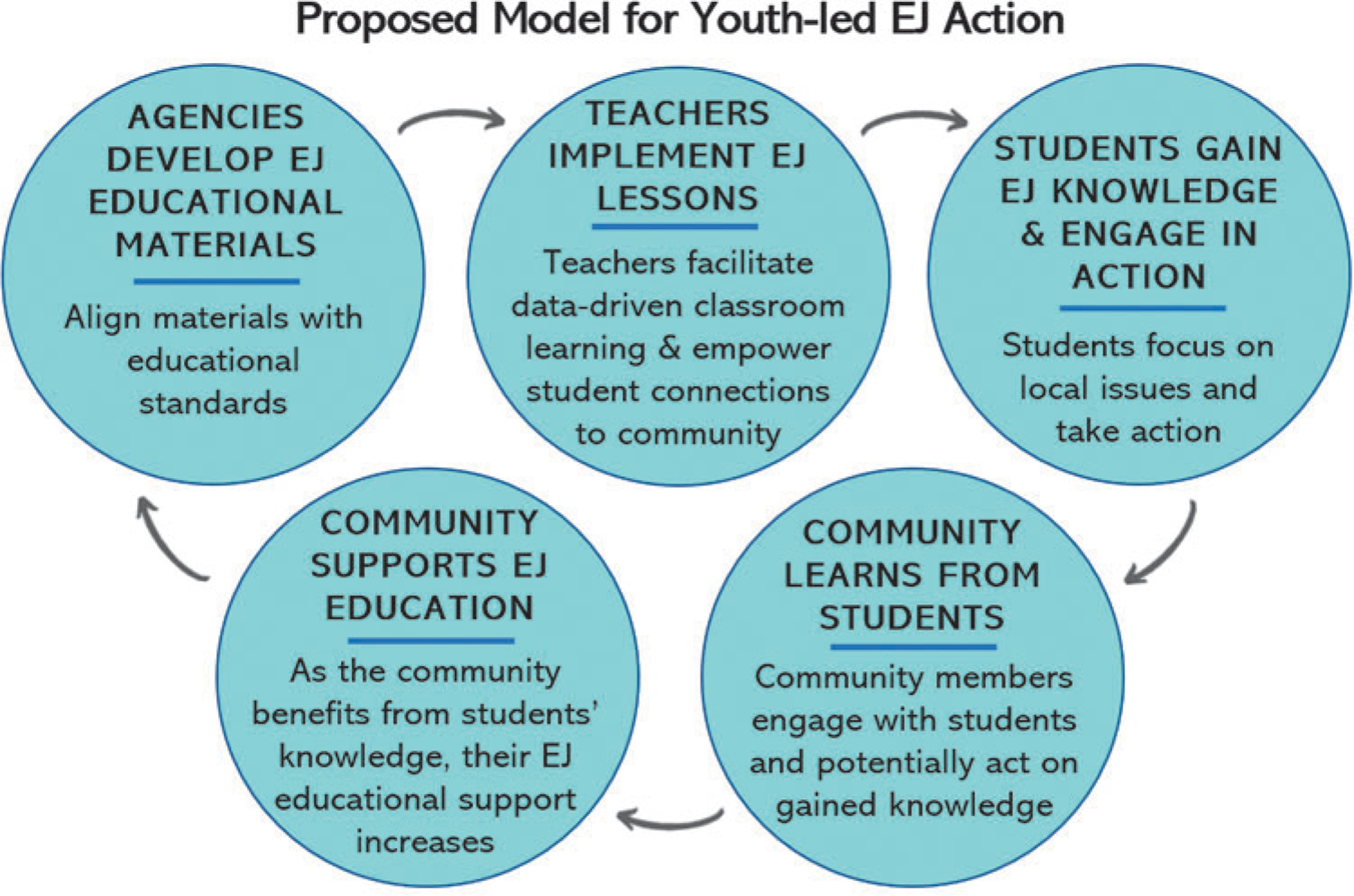
Proposed model for youth-led environmental justice action that has the potential to create several positive feedback loops, where students benefit from engaging communities while communities benefit from youth-led action. Adapted from the virtuous circle model in: Jenna M. Hartley *et al.*^10^ EJ, environmental justice.

In this article, we present the development of an EJ educational case study based on a collaboration between U.S. EPA’s Office of Environmental Justice and Office of Research and Development (ORD) and the Chesapeake Bay Foundation (CBF) (hereafter referred to as the “EPA Case Study”). Through the EPA Case Study, we acknowledge the overall lack of existing EJ-focused educational materials and state the need for such materials that utilize U.S. EPA’s mapping tools. We also describe the development and application of the EPA Case Study and share lessons learned for educational materials development from our perspective. We focus on the goal for the EPA Case Study to leverage the U.S. EPA’s mapping tools for data-driven local-level decision contexts, empowering young people to galvanize their families,^3^ communities,^4^ and even local policymakers toward informed environmental^5^ and potentially EJ policy considerations (Fig. 1).^6^ For example, young people have advocated in communities with EJ concerns, ranging from the Flint Michigan water crisis^7^ to initiatives tackling soil lead levels in Massachusetts^8^ and fighting for indigenous water rights as part of the International Indigenous Youth Council.^9^ As such, we conclude with a literature-backed call to action for continued federal support in the development of tools and educational materials that empower citizens of all ages while supporting informed decision making and policy that has the potential to result in real, lasting change.

## U.S. EPA’S MAPPING TOOLS

The EJSCREEN and EnviroAtlas^11^ online mapping tools were designed with various uses in mind, including to inform decision making and research. EJSCREEN was conceptualized in 2010 as an EJ mapping and screening tool to “provide EPA with a nationally consistent dataset and approach for combining environmental and demographic factors.”^12^ It has been used inside the EPA since 2012, was initially made public in draft form in 2015, and is updated annually. As a mapping tool, EJSCREEN compiles data from across the United States to identify vulnerable communities and areas with higher environmental burdens and combines those environmental and demographic data into EJ indexes.^13^ EnviroAtlas is a collection of online tools and resources, including an interactive mapping application, that was released in 2014 and has been updated in subsequent years.^14^ EnviroAtlas focuses on the concept of ecosystem services, or those benefits that humans naturally receive from the environment, their beneficiaries, and factors that may affect their provision. Although these two mapping tools vary in scope and design, they both serve to make national-scale environmental and demographic data freely available to the public and can inform EJ decision making, policymaking, and resource allocation for communities that potentially need them the most.^15^

Studies show that engaging stakeholders with mapping tools can make problems and solutions more relatable, thereby increasing support for environmental management actions where people live, work, play, and pray.^16^ EJSCREEN and EnviroAtlas are powerful tools that have the potential for being used toward such ends, as they are designed to empower anyone with an internet connection to be an informed decision maker. Citizens alike can easily incorporate environmental and demographic data from these tools to explore issues that matter to them, potentially influencing policy. In the face of a deeply divided nation that continues to struggle with an awareness of systemic and structural racism,^17^ simply providing these mapping tools is not enough. Providing relevant and robust EJ educational materials alongside spatial data is one necessary addition for mapping tools such as the U.S. EPA’s EJSCREEN and EnviroAtlas to make the tools more accessible and truly empower citizens.

The EJSCREEN and EnviroAtlas teams have made efforts to provide meaningful educational and training materials, including offering regular webinar trainings and guides. Both teams provide online training materials, a User Guide, and video tutorials^18^; in addition, EnviroAtlas provides fact sheets for every map layer, example use cases/case studies, and educational modules for students K-undergraduate. These educational resources have aided the use of these tools by local, state, and federal governments, as well as educators, students, and citizens. For example, middle school through graduate-level classroom teachers have recently used the EPA’s tools and accompanying materials, adapted for use with and without internet, generating student-focused conversations around EJ concepts and local issues.^19^ After introductory training, one Oregon professor had her students use the mapping tools to design their final class projects around local EJ issues of interest—some of those projects led to on-the-ground collaborations and action with local groups. Improving and developing additional EJ educational materials can only expand this type of usage and resulting impact.

Despite these educational efforts, the request to U.S. EPA from CBF for EJ-focused educational materials highlighted what many scholars, pedagogues, and researchers have cited for decades: there is an overall lack of EJ-focused educational materials,^20^ even though EJ themes fit easily into multiple STEM (Science, Technology, Engineering, and Math) subjects (e.g., mathematics,^21^ science,^22^ and engineering).^23^ We worked with CBF to meet their request, recognizing the broad historical exclusion of EJ-focused educational materials and buoyed by the collective voice of interdisciplinary scholars who have long suggested that this gap could be easily remedied.^24^

## ANSWERING THE REQUEST FOR EDUCATIONAL MATERIALS

In Spring 2020, members of EJSCREEN and EnviroAtlas collaborated with CBF in response to their request to incorporate U.S. EPA mapping tools and educational modules into their two summer EJ professional learning programs for educators. CBF is an independent conservation organization with a robust education and outreach program; their two voluntary summer EJ professional learning programs were each designed for ~30–35 K-12 educators. The collaboration to develop new EJ-focused educational materials was partially necessitated by the fact that in addition to a broad lack of EJ educational materials, the U.S. EPA also did not offer EJ-focused educational materials (at the time of writing) or mention EJ on its webpage “Lesson Plans, Teacher Guides and Online Environmental Resources for Educators.”^25^ To begin to fill that gap and respond to CBFs request for educational materials containing real-world data, the EPA Case Study team adapted an existing EnviroAtlas educational case study. The development team chose a case study approach with role-playing exercises because case studies have been shown to empower young people as decision makers capable of problem-solving,^26^ and empowerment is “an important tenet of social justice education … so that students can engage in learning that aims to rectify social injustices.”^27^

In addition to serving students, local-level experiential education activities such as role-playing have been proposed by multiple scholars as one way to connect classrooms to communities^28^ and support EJ education efforts.^29^ Furthermore, a major component of EJ work is collecting data and information about local concerns that are often overlooked or ignored by governing bodies and then bringing those concerns to light in analysis and decision-making contexts.^30^ Therefore, engaging young people via education as part of the process in local-level exploration of data and potentially subsequent participatory action research^31^ can contribute meaningfully to real-time EJ work while also contributing to meeting scientific educational standards within the classroom. For these reasons, CBF desired materials based in experiential learning that would get tools, resources, and information directly into the hands of teachers who could then use them for meaningful student empowerment.

## CASE STUDY

**FIG. 2.**
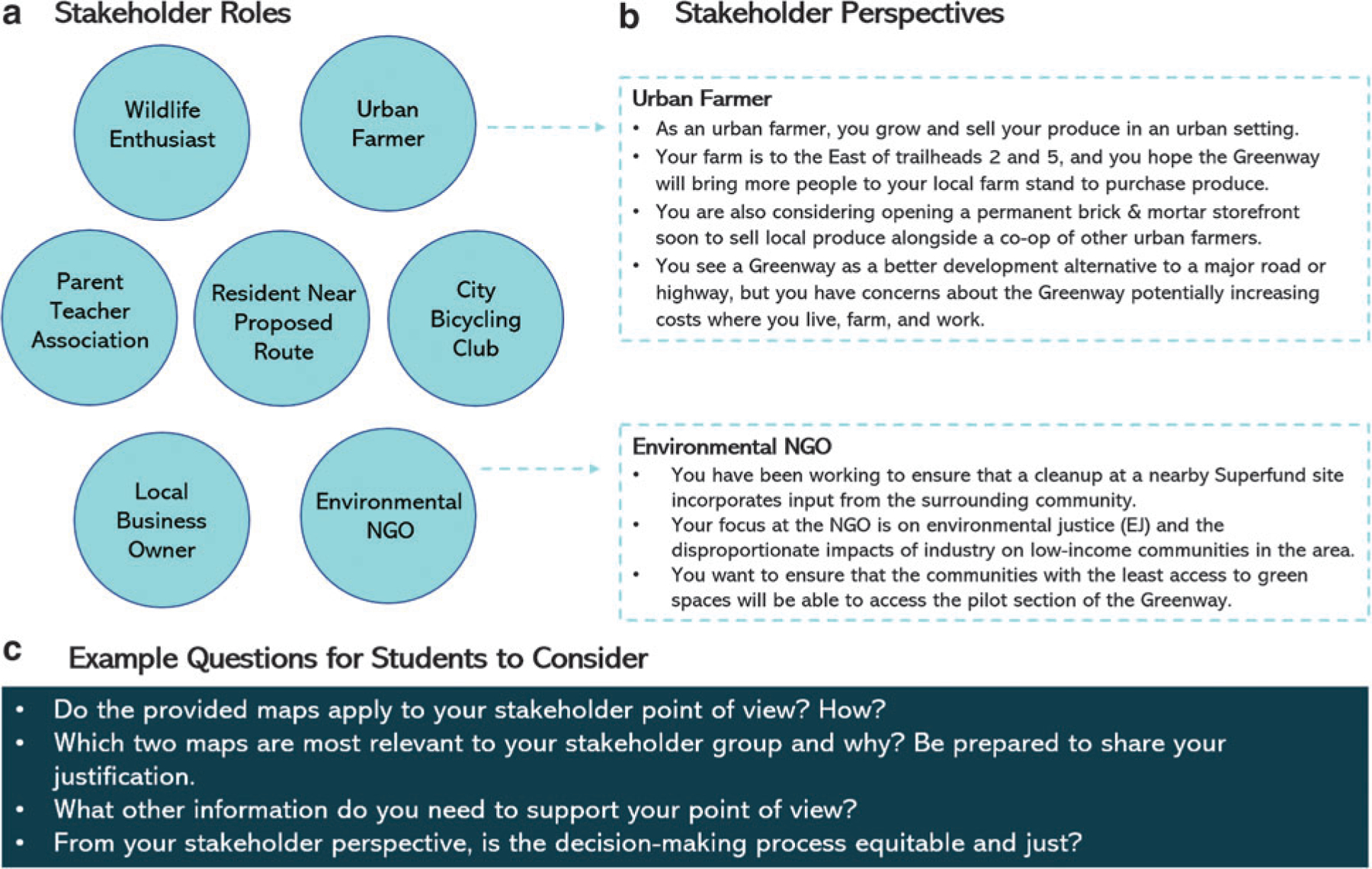
All stakeholder roles (**a**) and examples of two stakeholder perspectives (**b**) that students are asked to use toward guiding their decision making in the EPA Case Study. Example questions are shown here (**c**) and additional guiding questions are provided in the EPA Case Study. EPA, Environmental Protection Agency.

**FIG. 3.**
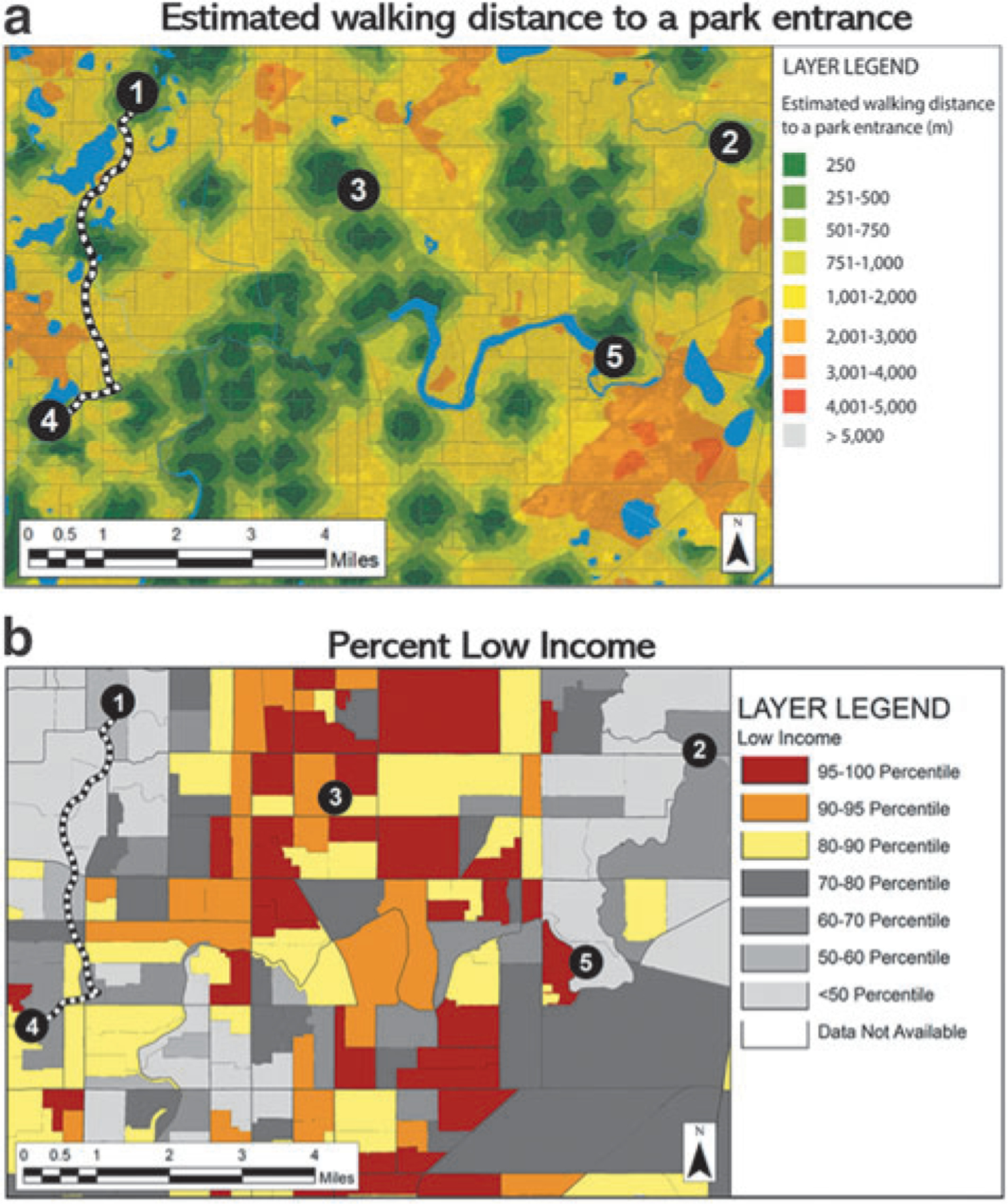
Samples of two community maps from the EPA Case Study demonstrating available data and the proposed trailheads for the greenway. (**a**) Map from U.S. EPA’s EnviroAtlas showing the estimated walking distance to a park entrance. (**b**) EJSCREEN map showing the percentage of households where the household income is less than or equal to twice the federal “poverty level.”

Guided by the goal of developing EJ-oriented educational materials that empower local decision making, the EPA Case Study was designed as a first activity of a planned series to be used by educators and practitioners. The case study creates a scenario where a city council is hosting a community town hall to decide on the location of a proposed greenway, or a network of trails connecting destinations in the city. Participants are assigned different stakeholder roles with associated descriptions (sample roles shown in Fig. 2) and given a set of community maps generated from EJSCREEN and EnviroAtlas (sample maps shown in Fig. 3). Participants reflect on their given stakeholder roles to make an individual decision on where they want to build the pilot section of the greenway, then in small groups work together to choose an overall route from their collective stakeholder perspective.

While the stated aim is for all the stakeholder groups to use the maps to inform how and where they each want to build a greenway from their stakeholder perspective and then have the group at large come to a consensus of where to build, the real take-home message is how nuanced, difficult, and even exclusionary such environmental decisions can be. It is through the smaller stakeholder group discussions and the large-group debate of intermixed stakeholders that the participants learn to use the map information to defend their positions and experience the real-world challenge of agreeing on an outcome that is not only acceptable, but equitable for everyone in the community. Role-playing exercises such as this lend themselves well toward teaching EJ^32^ as they encourage participants to reflect on multiple viewpoints other than their own^33^ since dominant groups are often blind to the struggles and perspectives of those who are marginalized.^34^ Furthermore, after the students complete the case study activity, they review maps from their own communities to explore issues similar to those addressed in the case study with an eye toward local-level solutions.^35^ As high school pilot participants noted, the activity helped them to better understand EJ from other perspectives while exposing them to types of statistics and data involved in such decision making.

Adapting the existing EnviroAtlas Greenway Case Study to include EJSCREEN data and EJ themes, with options available for classrooms with and without internet, took roughly 2 months. Most of this time was spent incorporating community demographics and characterizing EJ concerns. This required editing or adding background reading, stakeholder roles, maps, student handouts, educational standards, and additional virtual materials suited for interactive online learning. We solicited initial feedback from informal and formal environmental educators, CBF staff, and U.S. EPA staff (*n* = 9). Having incorporated their feedback, we piloted the new EPA Case Study in the two CBF-led summer programs with ~45 K-12 educators from across the Chesapeake Bay area in July and August 2020, who provided informal feedback during the session, and formal feedback via an approved, anonymous survey from EnviroAtlas (*n* = 20). We also piloted and refined the EPA Case Study with students: two classes of undergraduate students from Georgetown University (*n* = 20), middle and high school students in New York (*n* = 25), high school students in North Carolina (*n* = 45), and high school students in Michigan (*n* = 50), collecting formal feedback from their teachers (*n* = 3), one of whom voluntarily collected anonymous feedback from her students and shared it with us (*n* = 18). In total, 197 participants, including teachers, informal educators, and students, participated in pilot sessions between July 2020 and March 2021.

Once the EPA Case Study is made public in 2021, it can be used as a critical thinking activity for participants to incorporate EJ while using mapping tools and real-world data to work collaboratively toward local-level solutions. Especially since emerging studies support that youth voices can influence adult environmental attitudes in formal positions of local-level policy and decision making,^36^ we anticipate that outcomes from the EPA Case Study and ensuing EJ-focused educational materials could lead to local youth action and subsequent adult support.

## LESSONS LEARNED

Our lessons learned arose throughout the development process and informed our recommendations. To summarize, we recommend building an EJ case study from the ground up, allotting an appropriate amount of time for design and implementation of the activity, accommodating for virtual and in-person learning environments, and encouraging teachers to facilitate youth-led community engagement events if the students decide to take local action.

First, although scholars have identified that there has been a “missed opportunity” for educators to adapt existing educational materials with incorporation of EJ concepts^37^ and we followed such guidance to adapt the existing EnviroAtlas case study, we believe EJ issues would have been addressed more effectively had the case study been designed with EJ in mind from the outset. However, we benefited from collaborating across U.S. EPA offices and with an external partner to adapt our case study and conduct pilot tests with a variety of participants. Having multiple pilots, we delivered one session with a specific stakeholder role designed to incorporate the EJ perspective and one without to see how we could best facilitate and incorporate EJ concerns. Additionally, we had one pilot with EJ and equity concerns centered as part of the large group’s overall goal, and another where those goals were secondary to encouraging participants to stay firmly in their given stakeholder positions. Outcomes of this variation were mixed, but we saw that contrasting roles (examples of contrasting roles in Fig. 2) made for better debate and reflection of true circumstances. Consequently, when adapting a case study, we recommend that developers identify any available flexibilities to pilot different scenarios and allow for discussions of this nature to emerge.

Second, we recommend allotting an appropriate amount of time for design and implementation of the activity. While the original EnviroAtlas case study benefited from months of development, this team had a shorter time frame to rewrite it, include an EJ lens, and work with CBF to ensure that their needs were met. Additionally, meaningful refinements continued after the initial pilot period. Based on this, we recommend a minimum of 3 months, but ideally, up to a year to develop, refine, and pilot an EJ case study or module. Time was also a factor when designing the length of our own training sessions, and we chose to include important pedagogical strategies, such as map literacy, role-playing, and storytelling. We anticipate that classroom time is and will be a common limitation for teachers and educators, and we learned from pilot sessions that assigning preactivity tasks may alleviate this limitation. Many of the pilot participants who did not have preactivity tasks assigned, such as the maps, readings, and supporting materials, struggled with the maps upon seeing them for the first time during the group activity and suggested in their feedback to reduce the overall number of maps. On the contrary, students who did receive the pre-activity tasks seemed to have a better grasp of map literacy when later conducting the group activities. Accordingly, and in direct response to this feedback, we developed two versions with varying total maps to suit facilitators’ differing time restrictions related to map literacy.

Having ample time also allowed us to better incorporate elements of role-playing and storytelling, which we found important as those elements in educational settings may be one way to overcome participants’ barriers in perspective.^38^ Alternate voices and multiple perspectives are critical in environmental decision making, especially given that dominant voices can be the same ones that have oppressed at their worst and merely missed opportunities at their best.^39^ In reference specifically to educational materials based on mapping tools, educational developers should bear in mind that “statistics are important, but you can’t have statistics without stories, and vice-versa.”^40^ The same could be said for the freely available data from the U.S. EPA’s mapping tools—stories are essential to help describe the spatial data such that communities and policymakers are able to internalize it.^41^ We recommend an instructional design in which educators can ensure that there is adequate time allotted for students to explore map literacy, tell their own stories,^42^ assume roles as decision makers, state their opinions,^43^ and/or exert their own agency as empowered individuals.^44^

Given the recent shift in education to virtual settings, we recommend making accommodations for virtual and in-person learning environments. While adapting our existing in-person materials to incorporate EJ concepts, we were also able to incorporate commonly used digital teaching tools required for co-working and co-learning virtually in the time of COVID-19 school closures, and we encourage this for future material development. Last, following best practices on how best to engage children in civic action,^45^ we believe that if students choose to take subsequent local action in formal adult community settings, that they could be very impactful with the right amount of teacher support and facilitation.^46^ This support could range from the logistical facilitation of organizing student-led events to the preparatory support of helping students rehearse town hall speeches and provide feedback without imposing adult views.^47^ Not only does education research recommend these best practices,^48^ but we have witnessed the efficacy and positive youth-led community outcomes of this type of teacher support in the year since we piloted the materials with action-oriented teachers in EPA’s Region 8 (Great Lakes Region).^49^

## CALL TO ACTION

The U.S. EPA and other federal agencies can play a pivotal role in developing EJ-oriented educational materials, especially in keeping with recently stated goals.^50^ Incorporating EJ into educational pedagogy and “preparing present and future citizens capable of acting on a societal as well as a personal level”^51^ can have the potential for long-term and long-range environmental policy impacts. Indeed, empirical links between educational empowerment for young people and policy outcomes are emerging—recent research indicates that students transfer the environmental awareness they learn to their parents, sometimes even transcending socio-ideological differences.^52^ Examples of topics for which children have influenced their parents’ environmental actions or knowledge include watershed^53^ species,^54^ and energy^55^ conservation; water pollution^56^; flood resilience^57^; and climate change^58^; among others. There is no reason why this phenomenon of youth-led intergenerational learning could not also be true for the topic of EJ, especially as youth have been shown to prioritize and care deeply about environmental and social topics.^59^ No longer do the bounds of social justice participation begin and end with just casting a vote or signing a petition; young people empowered as student citizens^60^ are often found on the front lines of current affairs whether the methods are protesting, volunteering for organizations, online engagement,^61^ or directly contacting public officials.^62^ Accordingly, designing EJ curricula to encourage intergenerational learning and civic engagement may work to harness the enthusiasm and aptitudes of young people as well as provide all citizens with the tools and information needed to support them (Fig. 1).

The U.S. EPA’s freely available mapping tools, EJSCREEN and EnviroAtlas, have a unique opportunity to simultaneously communicate science and spur real change via motivated public engagement. Recognizing that providing data alone is insufficient,^63^ we contend that a multifaceted, intentional, and cross-agency approach by the U.S. EPA and other federal agencies would increase the potential for these data-driven mapping tools to empower EJ decision making. The EPA Case Study was a first attempt to address what has historically been excluded in educational spheres and use the EPA Case Study itself to advocate for more: more U.S. EPA collaborations, more EJ-focused educational materials, and more attention to developing these alongside the U.S. EPA’s mapping tools. In short, we are calling for increased EJ educational materials and EJ outreach around the U.S. EPA’s mapping tools by highlighting what has already been done (including a lack thereof), what work yet remains, and what kinds of positive impacts and social changes are possible in a future that heeds the call.
